# Coupled Evolution of Oil Content and Pore Structure of the Qingshankou Formation Shale, Songliao Basin, Northeastern China: Insights from Thermal Simulation Experiments

**DOI:** 10.1021/acsomega.5c00238

**Published:** 2025-05-29

**Authors:** Xuening Qi, Lianjie Tian, Kun He, Zhichao Yu, Minghao Wu, Jing Zhao

**Affiliations:** † Research Institute of Petroleum Exploration and Development, PetroChina, Beijing 100083, China; ‡ Key Laboratory of Petroleum Geochemistry, China National Petroleum Corporation, Beijing 100083, China; § School of Petroleum Engineering, 12412 Changzhou University, Changzhou, Jiangsu 213164, China

## Abstract

Oil content estimation and pore characterization of shale are crucial for identifying sweet spots in shale plays. Despite extensive research, the correlation between retained hydrocarbon characteristics and pore evolution in shale with thermal maturity remains controversial. This study employs an integrated approach, combining thermal simulation experiments with multiple analytical techniques, to investigate organic geochemical characteristics and pore structure evolution across different maturity stages. The results demonstrate that thermal maturity plays a significant role in evolution and synergistic changes in oil content and pore characteristics. Within the optimal maturity of 1.20%–1.60%, shale has three key favorable characteristics: (1) elevated gas-to-oil ratios, (2) enhanced fluidity of retained hydrocarbons, and (3) well-developed pore networks that facilitate efficient hydrocarbon. Combined with the geological conditions of the Songliao Basin, we identify that the shale oil in the Gulong Depression with an optimal maturity range of 1.20%–1.60% is the most promising exploration target.

## Graphical abstract

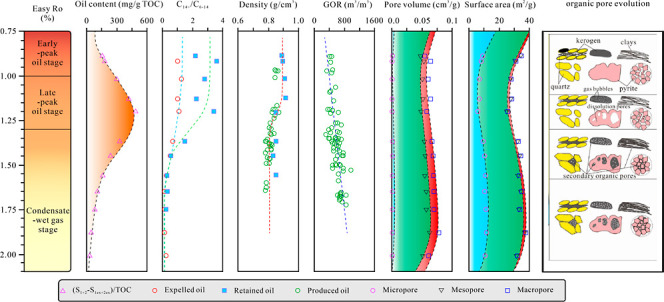

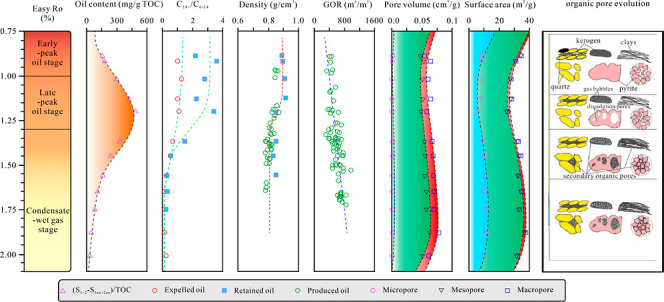

## 1 Introduction

In recent years, shale oil exploration in the Qingshankou Formation of the Songliao Basin has made significant breakthroughs, with estimated shale oil resources in the northern Songliao Basin of ∼151 × 10^8^ tons.^1,2^ According to the type and composition of the reservoir, continental shale oil is typically classified into interbedded thin sandstone-interbedded type, mixed type, pure shale type, etc.^3,4^ The Qingshankou shale oil in the Songliao Basin is a typical pure shale type, where oil is predominantly retained within clay-rich source rocks.^5^ Compared to other shale oil types, it is characterized by a high proportion of shale, high clay content, and small pore diameter.^5,6^ Consequently, the porosity characteristics and oil content are critical parameters for predicting sweet spots during the exploration and development of shale oil in the Qingshankou Formation.

Numerous studies have investigated the influence of thermal maturity, kerogen type, and organic matter content on pore variations^7–15^ and explored the interactions between hydrocarbon generation evolution and pore characteristic. Research on the pore evolution of samples using natural maturation sequences has demonstrated that maturity is the primary factor influencing changes in shale pore characteristics. The total pore volume significantly reduces from the immature to the late-mature stage, while postmature shale exhibits higher porosity and larger total pore volumes compared with late-mature shale.^14^ The increase in porosity may be attributed to the thermal conversion of kerogen into petroleum, which generates a carbon-rich residue and enhances porosity within the rock matrix.^16^ Furthermore, due to the rearrangement of pore structure and the formation of new pores during hydrocarbon generation, micropore volume gradually increases with maturity.^9^ In contrast, mesopore volume decreases from low to mature stages owing to compaction, while it subsequently increases again due to the transformation of organic matter into hydrocarbons at the overmature stage.^12^

Meanwhile, given the nonhomogeneous nature of shale samples and wider range of maturity, numerous laboratory thermal simulation experiments have been conducted to investigate the relationship between pore characteristics and shale hydrocarbon evolution. Chen and Xiao et al. have proposed that pore characteristics are influenced by organic matter content.^11^ In organic-rich shales, micropore and mesopore volumes exhibit an initial increase before declining. Beyond the maturity of 3.5%, the mesopore volume progressively increases with advancing maturation. In contrast, the specific surface area and pore volume of micro- and mesopores in organic-poor shales decrease with increasing maturity. Thermal simulation experiments suggest that the development of meso- and macropores after the oil generation peak primarily results from hydrocarbon expulsion. Although there is a close relationship between organic matter transformation and pore development, there is a lack of comprehensive geochemical evidence to demonstrate the correlation between the properties of hydrocarbons generated and pore characteristics.^17^ During the thermal evolution, the increase in micro-, meso-, and macropore results from further conversion of organic matter to hydrocarbons. At temperatures greater than 600 °C, pore size rearrangement leads to a decrease in micro- and mesopores (<10 nm) and an increase in macropores (>10 nm).^18^ Additionally, other studies have proposed that the pore volume and specific surface area initially decrease and then increase with thermal evolution.^19,20^

Although previous studies have explored the pore evolution in different types of shale across different regions, the coupled relationship between hydrocarbon expulsion, retention, and pore development during shale oil generation remains poorly understood. This knowledge gap hinders the selection of favorable exploration zones and accurate evaluation of shale oil resources. In this study, a closed system was employed to artificially mature low-maturity, organic-rich shale from the Qingshankou Formation, simulating time–temperature equivalent vitrinite reflectance (*R*o) values based on hydrocarbon generation kinetics, with experimental *R*o values ranging from 0.89% to 2.01% at intervals of 0.03%–0.17% (average interval of 0.1%). Detailed geochemical analyses of expelled hydrocarbon and retained hydrocarbon of the products were carried out along with low-temperature gas adsorption experiments on shales of various maturity stages. The simulation experiment approach mitigates the heterogeneity of natural shale samples and uses low-temperature spectroscopy to simulate high-maturity samples for a long time, as opposed to the high temperature in other studies. This low-temperature and long-duration simulation method provides a more precise representation of low-temperature, slow hydrocarbon generation under realistic geological conditions. Furthermore, a narrower *R*o interval was adopted to obtain detailed changes of oil content, hydrocarbon potential, and pore characteristics across a wide range of maturity sequences, from low maturity to overmaturity. This study aims to establish a coupled relationship between the hydrocarbon evolution and pore characteristics in Qingshankou Formation shale, providing valuable insights for the exploration and development of shale oil at different maturity stages.

## 2 Geological Setting

The Songliao Basin is one of the most petroliferous sedimentary basins in northeastern (NE) China, which is approximately 750 km long and 350 km wide with an area of ∼26 × 10^4^ km.^2,21^ The Songliao Basin is mainly subdivided into six structural units according to the current tectonic units: the Northern plunge zone, the Northeastern uplift zone, the Western slope zone, the Central Depression, the Southeastern uplift zone, and the Southwestern uplift zone (Figure 1a–c).^21,22^ The Central Depression, the main oil- and gas-producing region discussed in this study, comprises ten secondary structural units. The Cretaceous strata, i.e., Quantou (K_2_ *q*), Qingshankou (K_2_ *qn*), Yaojia (K_2_ *y*), and Nenjiang (K_2_ *n*) formations, are the primary source rock beds in the central depression.^23,24^ The K_2_ *qn* formation was deposited in a freshwater environment^24^ and is characterized by black shale interbedded with thin layers of siltstone, shell limestone, and dolomite. The total organic carbon (TOC) values of K_2_ *qn* shale vary from 1.0 wt % to 9.0 wt %.^1^ The organic matter maturity of the K_2_ *qn* shale in the Songliao Basin exhibits a broad range from 0.5% to 1.6%.^1,25^

**Figure 1:**
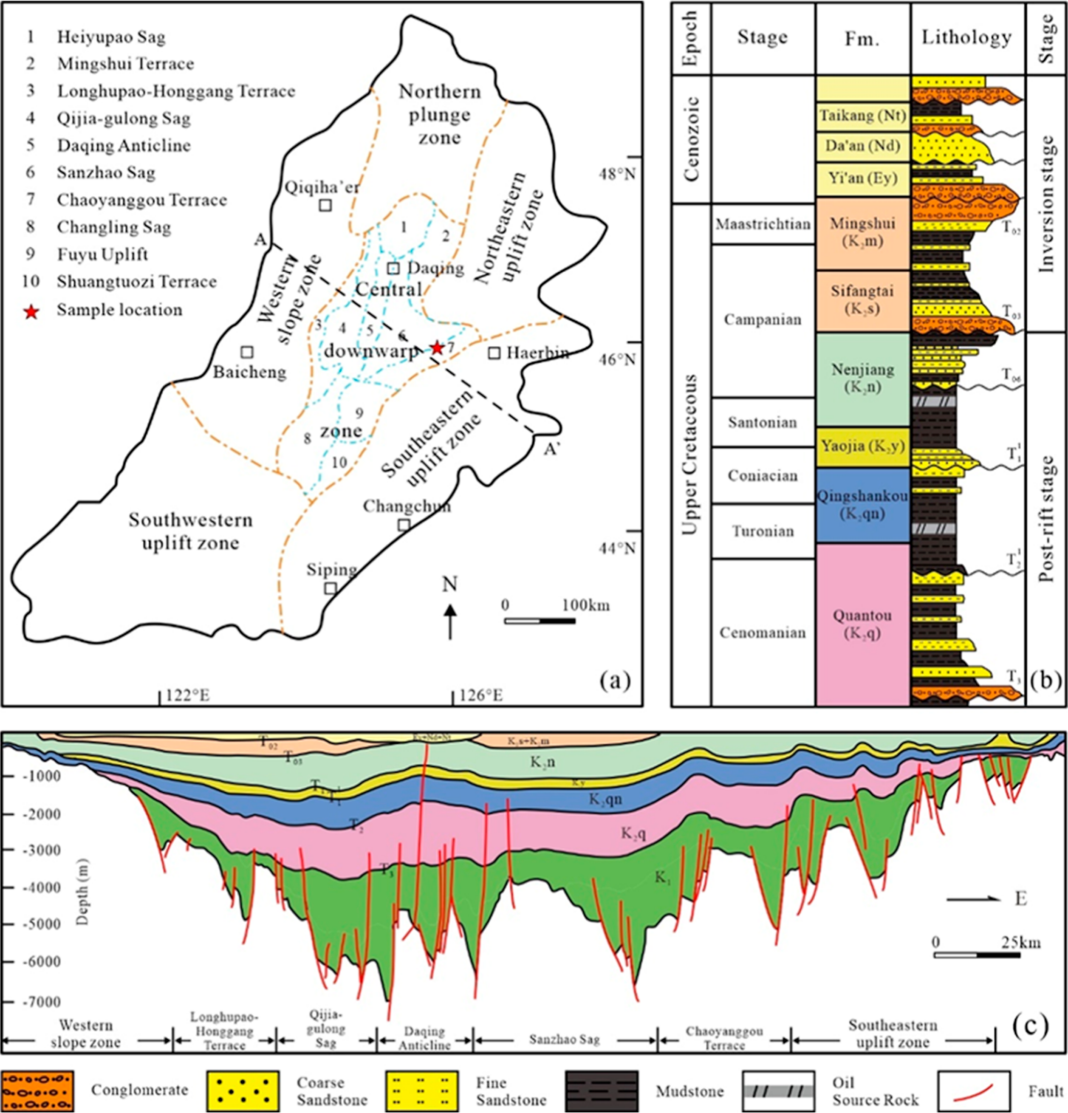
(a) Maps showing tectonic units of the Songliao Basin, NE China,^22,23^ and the location of core samples in this study; (b) stratigraphic column depicting the primary lithologies and evolutionary stages of the Songliao Basin;^22,23^ and (c) a section showing the stratigraphic structure of the Songliao Basin.^22,23^

## 3 Samples and Methods

### 3.1 Samples

The original shale samples were collected from the first member of the Qingshankou Formation (K_2_ *qn* ^1^) in Sanzhao Sag, which represents one of the secondary tectonic units within the Central Depression of the Songliao Basin. These selected samples exhibit a remarkably high TOC content of 4.87%. Rock-Eval pyrolysis analysis indicates the retained hydrocarbon (*S*_1_), pyrolyzed hydrocarbons (*S*_2_), carbon dioxide (*S*_3_), and peak temperature (*T*_max_) are 0.82, 42.84, 0.33, and 440 °C, respectively. Based on the hydrogen index (HI) and oxygen index values, this sample is characterized by Type I kerogen. The vitrinite reflectance is 0.70% *R*o, suggesting a relatively low maturity.

### 3.2 Experimental Methods and Procedures

The samples selected for the pyrolysis experiments underwent rigorous preparation procedures. Initially, they were carefully polished to remove any weathered surfaces, followed by thorough cleaning with deionized water. Subsequently, the samples were mixed to ensure homogeneity and crushed into grains ranging from 60 to 80 mesh. Before experimentation, all samples underwent a drying process in a vacuum oven at 80 °C for 24 h to eliminate moisture content from the rock particles. This rigorous preparation protocol ensured the integrity and reliability of the subsequent pyrolysis results.

#### 3.2.1 Closed Artificial Maturation System and Analysis Process

A closed anhydrous pyrolysis system equipped with a high-pressure pyrolysis apparatus developed by PetroChina Research Institute of Petroleum Exploration and Development was employed.^26^ Typically, 100 g of sample was loaded into a stainless-steel cylinder with an inner diameter of 34 mm. A pressure of 80 MPa was applied from one end of the cylinder along the axial direction to tightly compact the sample grains. Under this condition, given by elevated internal pressure associated with both hydrocarbon generation and increased temperature, a portion of the retained hydrocarbons naturally flowed out of the heated zone through a slim steel tube at the bottom of the cylinder. In this paper, the samples underwent pyrolysis using the same heating setup as in the closed system where the experimental conditions are much closer to natural geological settings, accounting for the accumulation or micromigration of shale oil within the shale.

The dried samples were divided into 14 fractions, each transferred to a series of stainless-steel tubes and sealed with a graphite pad. Pyrolysis was conducted at temperatures ranging from 330 to 420 °C and durations ranging from 2 to 8 days, and the equivalent reflectance (Easy%*R*o) was calculated using the methods proposed by Sweeney and Burnham.^27^ Each temperature point with a different duration time and its corresponding Easy%*R*o is presented in Table 1. The products of the simulation experiment included gas, expelled oil, and simulated samples (shale samples remaining after thermal simulation without extraction). Gas was collected using the salt-water displacement method after the experiment ended, and the furnace temperature dropped to room temperature. Expelled hydrocarbons refer to hydrocarbons washed from the inner walls of the cylinder and transfer lines with dichloromethane after each pyrolysis. The simulated samples were transferred to covered glass bottles and stored in a refrigerator to prevent the loss of volatile components before the experimental analysis. Extractable organic matter (EOM) was obtained by extracting the simulated samples in a Soxhlet extractor with dichloromethane (CH_2_Cl_2_) for 72 h. The solid samples after extraction were referred to as residues.

**Table 1:** Thermal Simulation Experiment Temperature and Time Corresponding to Easy%*R*o^a^

sample#	temperature (°C)	time (days)	Easy%*R*o
1	330	3	0.89
2	330	4	0.92
3	350	2	1.02
4	350	4	1.13
5	350	6	1.20
6	380	2	1.37
7	380	3	1.45
8	380	5	1.56
9	400	2	1.65
10	400	3	1.75
11	400	5	1.88
12	400	8	2.01

a “Temperature” refers to the temperature of the kettle after 2 h heating from room temperature, which is maintained at a constant temperature during the pyrolysis process. “Time” represents the time of thermal reaction of the original sample in the reactor under the temperature condition of step. Easy%*R*o was calculated using the method proposed by Sweeney and Burnham.^27^

Gas, expelled hydrocarbons, and chloroform asphalt were subjected to component analysis using gas chromatography (GC). TOC analysis for simulated samples and residues was performed by using a LECO-CS230 analyzer. Rock-Eval VI was used for the rock pyrolysis analysis of simulated samples and residues. Low-temperature gas adsorption analysis was performed by using a Micromeritics ASAP 2020 surface area analyzer. The experimental process is illustrated in Figure 2.

**Figure 2:**
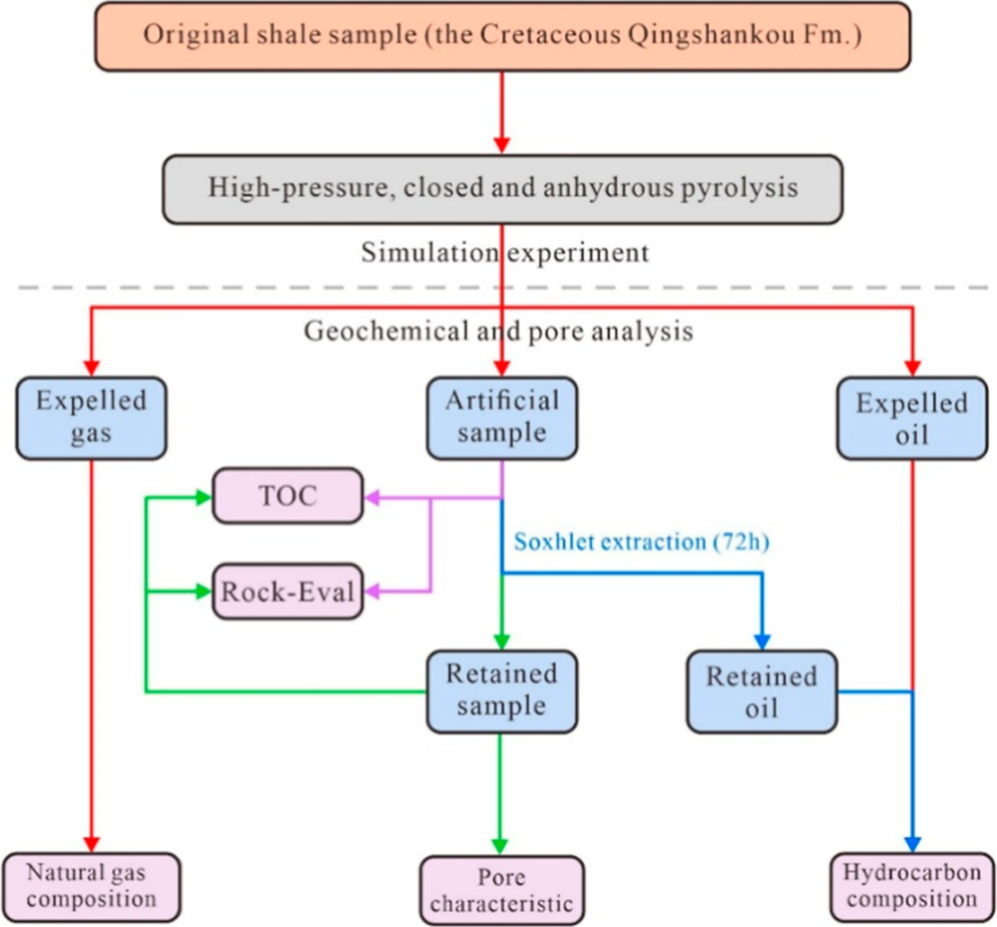
Flowchart illustrating the coupled relationship between oil content and porosity.

#### 3.2.2 TOC and Rock-Eval Analysis

A portion of the pyrolyzed sample without solvent extraction and the residual samples’ postchloroform extraction were ground to a ∼200 mesh powder. The powder weighing between 30 and 100 mg was washed using 5 mol of HCl four times and pure water to remove inorganic carbon, such as carbonate. Subsequently, the washed samples were dried in a vacuum oven at 80 °C for 4 h. The dried sample was then placed in an oxidizing furnace of the LECO-CS230 analyzer. The temperature of the infrared light source in the detector was maintained at 850 °C.

The sample underwent Rock-Eval VI analysis to measure *S*_1_, *S*_2_, *T*_max_, and TOC. The pyrolysis temperature was programmed as follows: the sample was initially heated from room temperature to 300 °C, held for 3 min, and then heated to 850 °C at a rate of 50 °C/min.

#### 3.2.3 Analytical Methods of Pyrolyzed Products

The gaseous products were categorized into nonhydrocarbon gases (CO_2_, N_2_, and H_2_) and hydrocarbon gases (C_1_–C_5_). Compositional analysis was conducted using an Agilent 7980 A GC system equipped with five valves, six columns, dual thermal conductivity detectors (TCDs), and a single Flame Ionization Detector (FID). The Poraplot Q column (length 30 m × 0.25 mm × 0.25 mm film thickness) was utilized for separation. The temperatures of the inlet, TCD, and FID were set at 150 °C, 200 °C, and 250 °C, respectively. The oven temperature was initially maintained at 35 °C for 5 min, then increased from 40 to 70 °C at a rate of 3 °C/min, followed by a further increase from 70 to 200 °C at 5 °C/min, and held constant at 200 °C for 20 min. Helium served as the carrier gas for the FID and the first TCD, responsible for detecting C_1_–C_4_ hydrocarbons and N_2_, CO_2_, while nitrogen was employed as the carrier gas for the second TCD to detect H_2_.

GC analysis of the expelled oil and residue oil was conducted using an Agilent 7890 GC interfaced with an FID. An elastic quartz capillary column (length of 50 m × film of 0.25 mm) coated with HP-PONA was employed, and nitrogen was used as the carrier gas at a constant flow rate of 1.0 mL/min. The GC oven temperature was programmed to increase from 35 to 150 °C at a rate of 4.5 °C/min. This analytical setup facilitated detailed analysis of the hydrocarbon composition of both the expelled and residual oils.

#### 3.2.4 Low-Temperature N_2_ and CO_2_ Physisorption Analysis

Low-temperature CO_2_ and N_2_ adsorption experiments were conducted by using a Micromeritics ASAP 2020 surface area analyzer to measure total porosity. Before the N_2_ and CO_2_ experiments, 3–5 g of the residual samples from step 3.2.1 (postextraction) was utilized for more accurate quantitative characterization of the sample pores.^28–31^ To remove residual gas impurities, the samples were degassed in a vacuum chamber at 150 °C for 6 h. CO_2_ adsorption was employed to obtain information on micropores (diameter < 2 nm), while N_2_ adsorption was used to detect mesopores (diameter 2–50 nm) and macropores (diameter 50–300 nm).^32^ For CO_2_ adsorption, measurements were conducted at 273.0 K, with an adsorption equilibrium time of 45 s and a relative pressure range of *P*/*P*_0_ 0.00006–0.03, with adsorption continued for at least 10 h to obtain the CO_2_ adsorption curve. The samples were subjected to N_2_ adsorption–desorption isotherm measurements at a liquid nitrogen temperature of 77.35 K, with an equilibrium time interval of 30 s and a relative pressure range of 0.005–0.998 (*P* for equilibrium pressure and *P*_0_ for saturation pressure).

Micropore volume and pore size distributions were determined using density functional theory, while the specific surface area was obtained using the Dubinin-Astakhov method. Mesopore and macropore volume and pore size distributions were determined using the Barrett–Joyner–Halenda method.^33^ Specific surface area was determined using the Brunauer–Emmett–Teller model.^34,35^

## 4 Results

### 4.1 TOC and Rock-Eval Characteristics

The TOC values of both the unextracted and postextracted samples gradually decrease with thermal evolution until the *R*o reaches 1.65%, after which it stabilizes at around 1.80 wt % (Figure 3a). Compared with unextracted samples, postextracted samples generally have lower TOC values, stable values, and near-zero *S*_1_ values (Figure 3b).

**Figure 3:**
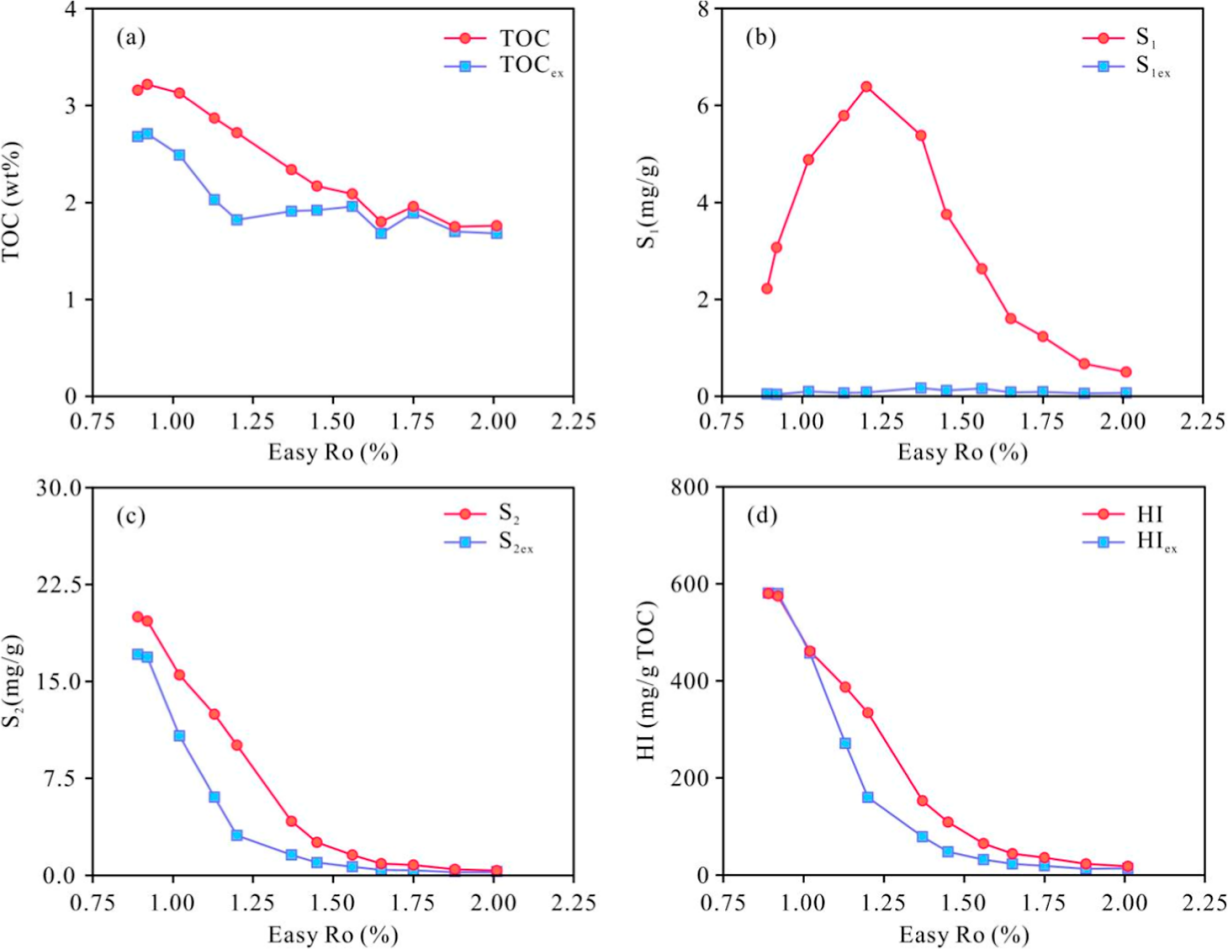
Variations of TOC and pyrolysis parameters in shale with maturity. (a) TOC; (b) *S*_1_; (c) *S*_2_; and (d) HI.

The *S*_1_ values of unextracted samples increase rapidly with rising maturity, with a peak value of 6.39 mg/g at an Easy%*R*o of 1.20%. The *S*_1_ values of postextracted samples show unobvious variations with increasing maturity (Figure 3b). The trends in *S*_2_ for unextracted and postextracted samples differ slightly from those of *S*_1_ and TOC with increasing maturity. In both unextracted and postextracted samples, the variations in TOC, *S*_2_, and HI index follow a consistent trend with increasing maturity, with all three parameters exhibiting a continuous decrease (Figure 3a,c,d).

### 4.2 Hydrocarbon Composition Characteristics

#### 4.2.1 Gas Composition Characteristics

The variations in the content of hydrocarbon gases (C_1_–C_6_) and nonhydrocarbon gases (i.e., carbon dioxide, nitrogen, and hydrogen) in the simulated gas products are shown in Figure 4a. When Easy%*R*o is less than 1.2%, the relative contents of nonhydrocarbon gases exceed those of hydrocarbon gases. As Easy%*R*o exceeds 1.2%, the relative contents of hydrocarbon gases increase steadily, exceeding those of nonhydrocarbon gases and eventually stabilizing close to 60%. When Easy%*R*o is >1.56%, hydrocarbon gases are predominant.

**Figure 4:**
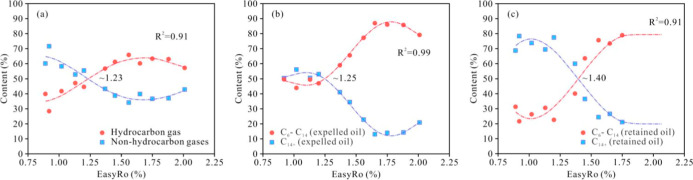
(a) Variations in hydrocarbon and nonhydrocarbon gases; (b) variations in C_6_–C_14_ and C_14_^+^ of expelled oil; and (c) variations in C_6_–C_14_ and C_14_^+^ of retained oil.

#### 4.2.2 Expelled Oil Composition Characteristics

The expelled oil composition is categorized into two fractions based on molecular weight: C_6_–C_14_ and C_14_^+^. When Easy%*R*o is less than 1.20%, the expelled oils contain approximately equal proportions of C_6_–C_14_ and C_14_^+^ (Figure 4b). Additionally, during the thermal evolution stage, C_14_^+^ is typically the predominant hydrocarbon, with the distribution of C_6_–C_14_ saturates displaying a “V”-shaped pattern in the gas chromatograms (Figure 5-1a,b). As Easy%*R*o exceeds 1.20%, the relative content of C_6_–C_14_ in the expelled oil increases rapidly, reaching a stable and high value of 85% at Easy *R*o of greater than 1.65%, while the content of C_14_^+^ has gradually declined to about 13% during this stage. As shown in Figure 5-1a–e, the main carbon peak of the expelled oil gradually shifts from C_15_ to C_5_.

**Figure 5:**
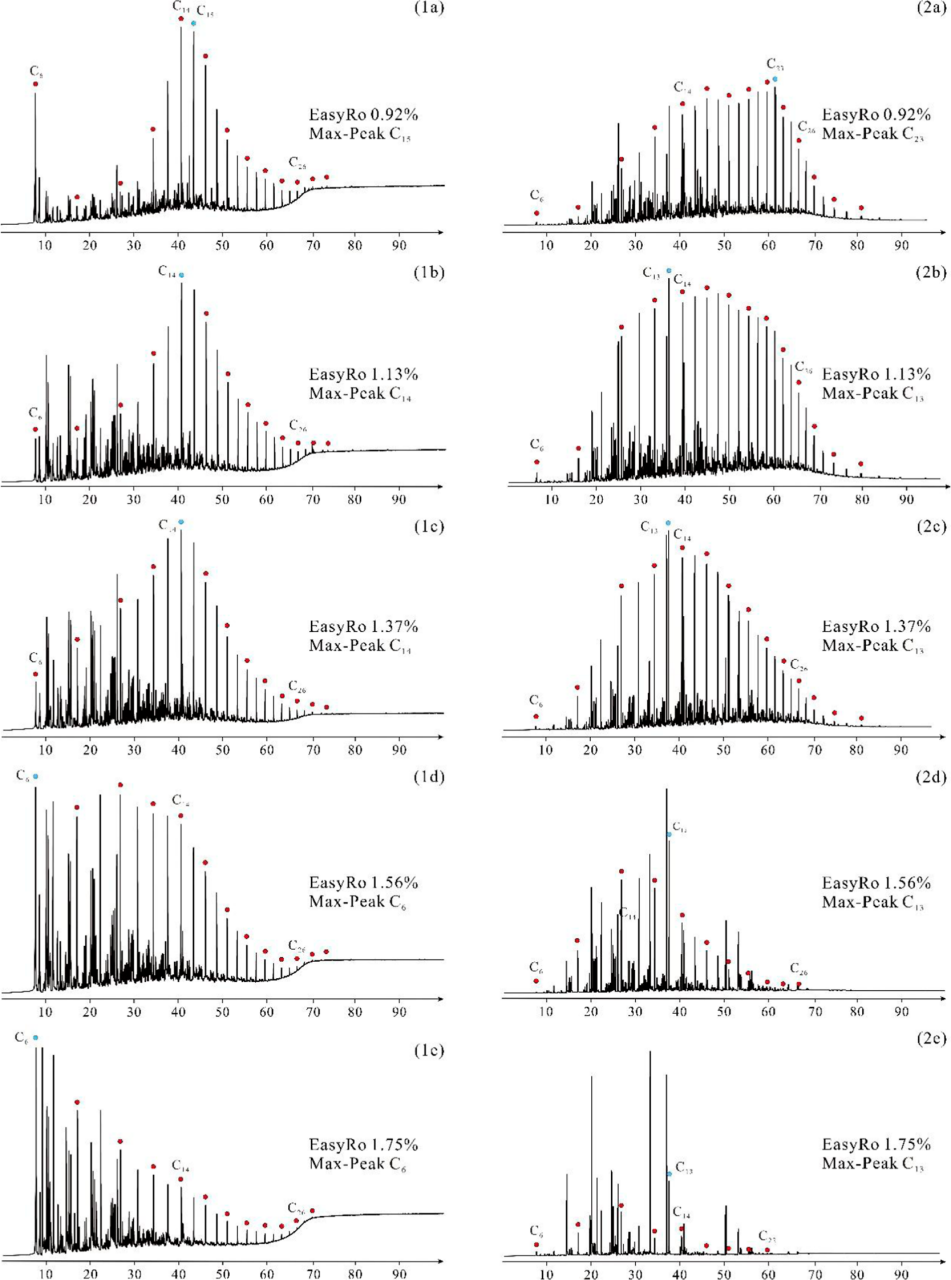
Gas chromatogram of expelled oil (left or sequence 1) and retained oil (right or sequence 2). Max-peak carbon is indicated by a blue dot; even-numbered carbons, including C_6_, C_14_, and C_26_, are marked with red dots.

#### 4.2.3 Retained Oil Composition Characteristics

Similar to the expelled oil, the compositions of the retained oils are also divided into two parts: C_6_–C_14_ and C_14_^+^ based on the molecular weight. When Easy%*R*o is greater than 1.20%, the percentage of C_6_–C_14_ hydrocarbons remains relatively stable and low (Figure 4c), ranging from 22.60% to 31.27%. The carbon number distribution of saturated hydrocarbons in the retained oil mainly ranges from C_14_ to C_30_ (Figures 5-2a,2b). When Easy%*R*o is in the range of 1.20%–1.75%, the relative content of C_6_–C_14_ in the retained hydrocarbons rapidly increases and is stable after an Easy%*R*o of 1.56%, with C_6_–C_14_ accounting for 75% of the total retained hydrocarbons. Within this maturity range, the forward shift of the maximum carbon number of *n*-alkanes and the decreased content of C_14+_ hydrocarbons are observed (Figure 5-2c,d). As Easy%*R*o exceeds 1.75%, the relative content of C_6_–C_14_ hydrocarbons gradually decreases, and the gas chromatograms exhibit a scattered distribution (Figure 5-2e). Therefore, with the increase in thermal maturity, the relative content of C_14_^+^ and C_6_–C_14_ hydrocarbons exhibits opposite trends (Figure 4c).

### 4.3 Low-Temperature CO_2_ and N_2_ Adsorption of Shale

#### 4.3.1 CO_2_ Adsorption Isotherms

The low-temperature CO_2_ adsorption isotherms exhibit Type I (b) behavior, which is characteristic of microporous materials.^35^ At a relative pressure (*p*/*p*_0_) of approximately 0.03, the maximum of the CO_2_ adsorption capacity ranges from 0.79 to 1.53 cm^3^/g across all samples, while the minimum of the CO_2_ adsorption volume is observed at an Easy%*R*o of 1.20%. In the postextracted samples, as Easy%*R*o increases from 0.71% to 1.20%, the CO_2_ adsorption volume gradually decreases. When Easy%*R*o is greater than 1.20%, the CO_2_ adsorption volume increases. When the Easy%*R*o is in the range 1.45%–1.88%, the CO_2_ adsorption volume increases with increasing maturity, followed by a decrease in the CO_2_ adsorption volume as maturity further proceeds.

#### 4.3.2 N_2_ Adsorption Isotherms

Low-temperature N_2_ adsorption isotherms of simulated shale samples show similar trends between the adsorption and desorption isotherms. According to the International Union of Pure and Applied Chemistry classification scheme for adsorption curve types (Figure 7-1a–c; 2a–d),^34^ the isotherms are Type IV and Type H_3_ hysteresis loops, indicating that the pore size distribution of these samples is predominantly mesoporous.^35^ The hysteresis behavior further indicates that the pore structure may consist of fissure-like pores within aggregates of platy particles (Figure 7).^36,37^

**Figure 6:**
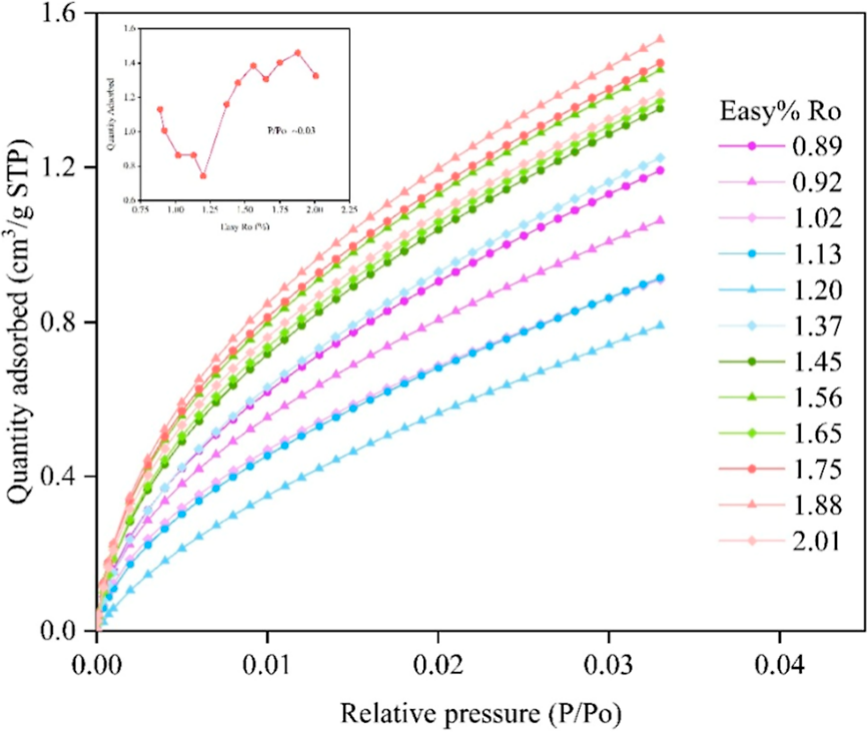
CO_2_ adsorption isotherm curve of the shale samples at different thermal maturity stages.

**Figure 7:**
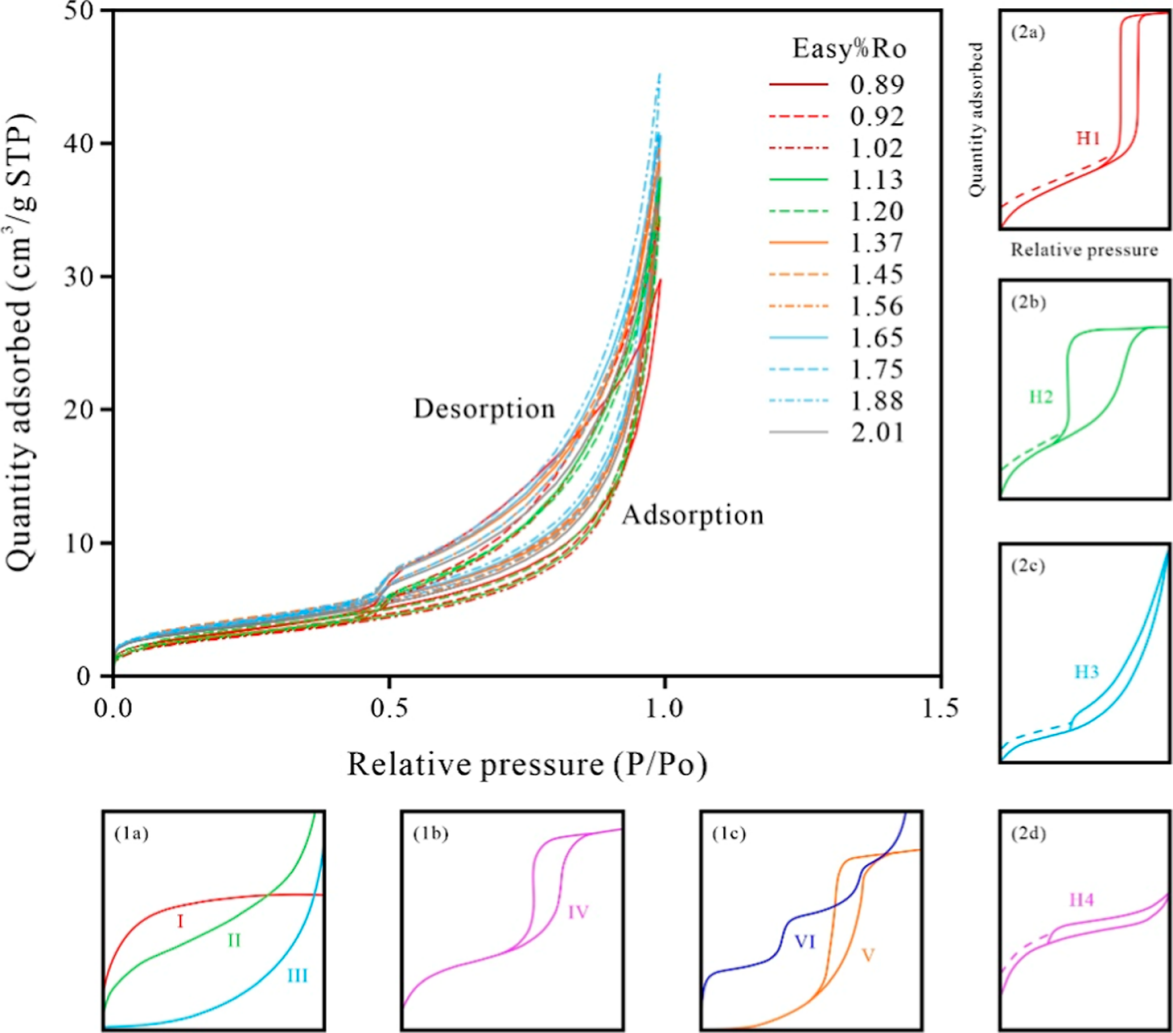
N_2_ adsorption–desorption isotherms for samples with different thermal maturity stages. STP stands for standard temperature and pressure.

#### 4.3.3 Pore Volume, Pore Surface Area, and Pore Size Distribution

The micro-, meso-, and macropore volumes of the simulated samples across different maturity stages range from 0.0006 to 0.0018 cm^3^/g (average 0.0013 cm^3^/g), 0.0465 to 0.0614 cm^3^/g (0.0527 cm^3^/g), and 0.0073 to 0.0156 cm^3^/g (0.0119 cm^3^/g), respectively.

The micropore volume of the simulated samples decreases from 0.012 cm^3^/g to 0.006 cm^3^/g as maturity increases from 0.70% to 1.20% Easy%*R*o. However, as maturity continues to increase from 1.20% to 2.01% Easy%*R*o, the micropore volume increases from 0.013 to 0.0018 cm^3^/g (Figure 8a). The changes in pore volume and surface area of the micro-, meso-, and macropores are distinctly different with increasing maturity (Figure 8). The pore volume and surface area of the micropore decrease from 0.012 to 0.006 cm^3^/g, and from 9.34 to 6.21 m^2^/g, respectively, as maturity increases from 0.70% to 1.20% Easy%*R*o. However, both the micropore volume and surface area increase as maturity increases from 1.20% to 2.01% Easy%*R*o (Figure 8a,b). In contrast, the mesopore volume shows an overall ascending trend as maturity increases from 0.70% to 2.01% Easy%*R*o (Figure 8c). The mesopore surface area sharply decreases with maturity between 0.70% and 1.20 Easy%*R*o and then increases again as maturity continues to rise (Figure 8d). The pore volume and surface area of macropores exhibit a continuous increase with the thermal maturity evolution (Figure 8e,f). The pore volume and surface area for all pore types decrease when the maturity exceeds 1.88% Easy%*R*o (Figure 8).

**Figure 8:**
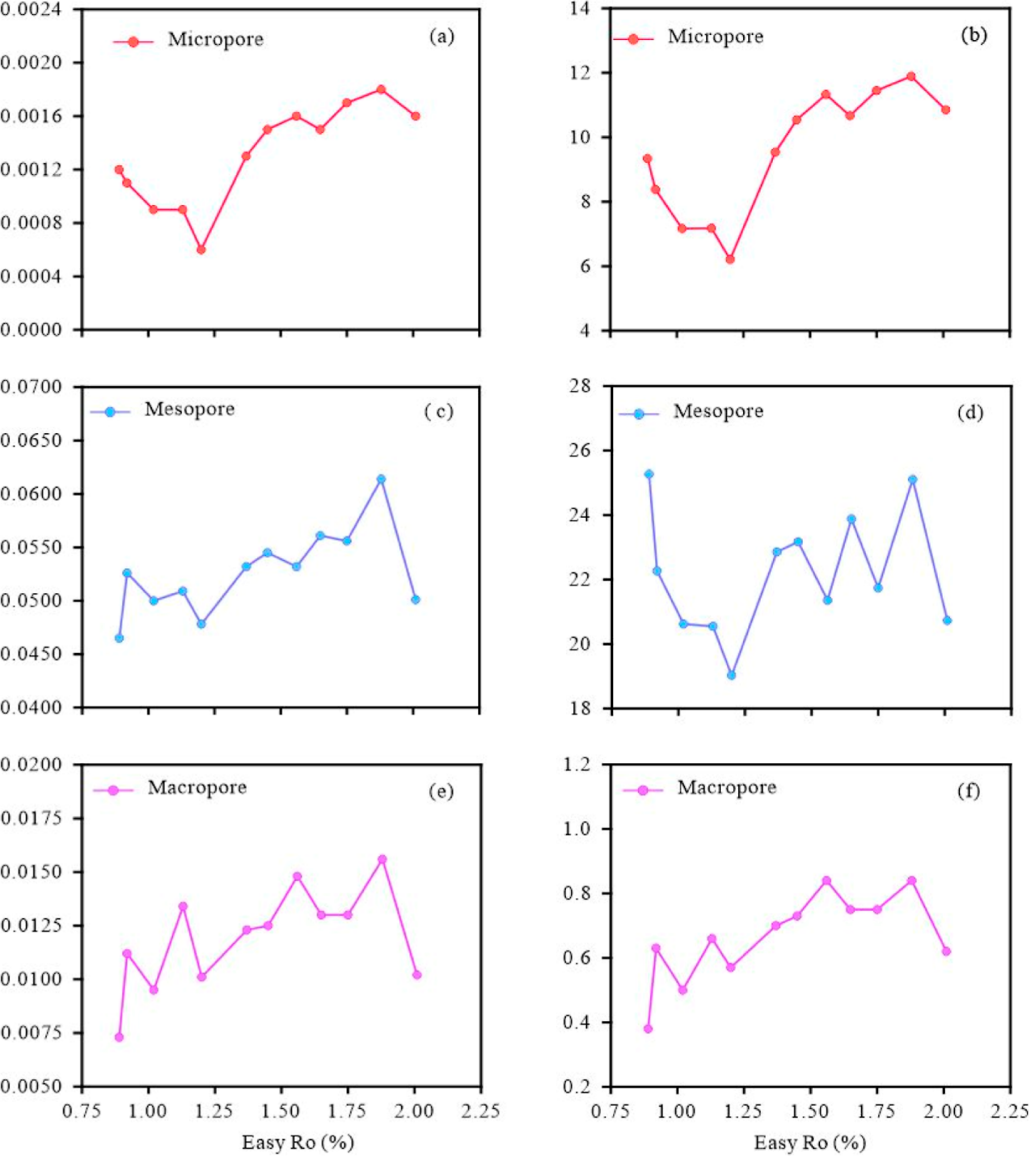
Variations in the pore volume and surface area of nanopores of the samples with increasing maturity. (a) Micropore volume; (b) micropore surface area; (c) mesopore volume; (d) mesopore surface area; (e) macropore volume; and (f) macropore surface area.

The shale samples are predominantly composed of micropores, followed by an approximately equal proportion of mesopores and macropores. Notably, the percentage of macropores in shale samples with high maturity (>1.65% Easy%*R*o) is higher than that in samples with low maturity (<1.65% Easy%*R*o).

## 5 Discussion

### 5.1 Generation and Evolution of Hydrocarbons

With increasing thermal maturity, the content and composition characteristics of both expelled and retained hydrocarbons undergo significant changes (Figures 4 and 5). In the oil generation window (0.89%–1.20% Easy%*R*o), hydrocarbons are primarily generated through thermal degradation of kerogen in the Qingshankou Formation Shale.^38^ During this period, nonhydrocarbon gases are dominant, while the content of hydrocarbon gases increases slowly (Figure 4a). Both expelled oil and retained oil are mainly composed of heavy components (C_14_^+^) (Figure 4b,c). The increase in *S*_1_ value (Figure 3b) indicates that trapped hydrocarbons with high C_14_^+^ content in shale exhibit poor fluidity.

From the oil generation to hydrocarbon cracking (1.02%–1.56% Easy%*R*o), the rate of TOC reduction is at its peak. Based on the difference in TOC values of unextracted and postextracted samples, and the change in *S*_1_, hydrocarbon generation within this maturity range can be divided into three stages: In Stage 1 (1.02% ≤ Easy%*R*o < 1.20%), the difference in the TOC values of unextracted and postextracted shale increases with increasing maturity, *S*_1_ increases with maturity, while *S*_2_ decreases, and the difference in the *S*_2_ of unextracted and postextracted shale is maximized (Figures 3b,c). *S*_2_ contributes the most to the composition of the retained hydrocarbons (Figure 3c). Compared with the maturity of less than 1.02% Easy%*R*o, the thermal degradation of kerogen becomes dominant, accompanied by the cracking of some hydrocarbons and an increase in the proportion of gas within the generated hydrocarbons. This process enhances the mobility of the hydrocarbons. In Stage 2 (1.20% ≤ Easy%*R*o ≤ 1.37%), the stage is dominated by the thermal cracking of kerogen, leading to the formation of large amounts of natural gas and light oil. The maturity of the peak hydrocarbon generation is 1.20% Easy%*R*o,^38^ exceeding the *R*o values (around 0.60%–0.80%) of the hydrocarbon generation peak in the Member IV of the Shahejie Formation of the Jiyang Depression and the Lower Ganchaigou Formation of the Qaidam Basin.^39,40^ The high clay mineral content in the Gulong shale may be one of the reasons for the delayed hydrocarbon generation peak. The interaction between clay minerals and organic matter, especially the adsorption of organic compounds on the surface of clay minerals, increases the activation energy required for hydrocarbon generation, thereby delaying the transition to the oil peak.^41,42^ Given organoclay composites in shale, clay minerals can either catalyze or inhibit the pyrolysis of organic matter.^43,44^ In shallow-buried shale where montmorillonite-to-Illite transformation remains incomplete, montmorillonite exhibits catalytic or inhibitory effects on hydrocarbon generation processes.^42,43^ Thus, this is a very complex question that requires further exploration. Additionally, the saline environment of the Shahejie Formation shale and the Lower Ganchaigou Formation shale may have promoted hydrocarbon generation, leading to an earlier hydrocarbon generation peak.^40^ In Stage 3 (1.37% < Easy%*R*o ≤ 1.56%), *S*_1_ begins to decline, accompanied by the initial cracking of heavy hydrocarbons, which results in an increased natural gas production and a higher gas–oil ratio compared with Stage 2. Thus, the shale oil mobility is optimal in Stage 3. Subsequently, when the maturity exceeds 1.56%, the rate of TOC decline slows down. During this stage, hydrocarbon thermal cracking becomes the dominant evolution, resulting in a higher gas/oil ratio compared with Stage 3. Although the C_6_–C_14_/C_14_^+^ ratio is high within this range, the amount of hydrocarbons is very low.

EOM is used to evaluate the total oil content of shale.^3,45^ Additionally, Jarvie et al. proposed that the retained hydrocarbons exist in the form of *S*_2_ during rock pyrolysis. The difference between *S*_1_ and *S*_2_ of unextracted and postextracted shale can reflect the total oil content.^3^ The difference between the EOM and the pyrolysis parameters of unextracted and postextracted shale shows that the pyrolysis parameters are higher than EOM, especially in the stage of Easy%*R*o of greater than 1.20% (Figure 10a). Compared to actual shale samples, the experimental samples were stored in a refrigerator to minimize the loss of hydrocarbons.^1,46^ However, the extraction process of the EOM results in the loss of many light components. Within the maturity range of 1.20%–1.56%, the C_14_^+^ fraction of the retained hydrocarbons decreases (Figures 5 and 10b), indicating the loss of a large amount of light component. Site-sealed pressure core experiments also show that there is a large number of volatile light components in the retained hydrocarbons of the Gulong shale, and the amount of light component is lost during conventional pyrolysis analysis.^1^ Considering the high content of light components in shale oil, the difference in pyrolysis parameters of unextracted and postextracted shale is accurate for quantifying the total oil content of the Gulong shale. At the peak of oil generation (Easy%*R*o = 1.2%), the total oil content is approximately 500 mg/g (Figure 10a), which is slightly lower than that in the gold tube pyrolysis experiment due to the release of a small number of hydrocarbons.^47^ This results in a total oil content that is slightly lower than 600–800 mg/g observed in experiments of the low-maturity Qingshankou Formation shale. However, it is 1–2 times higher than the actual oil content of 200–400 mg/g.^48^ Therefore, the Qingshankou Formation shale can provide a material basis for shale oil accumulation.

**Figure 9:**
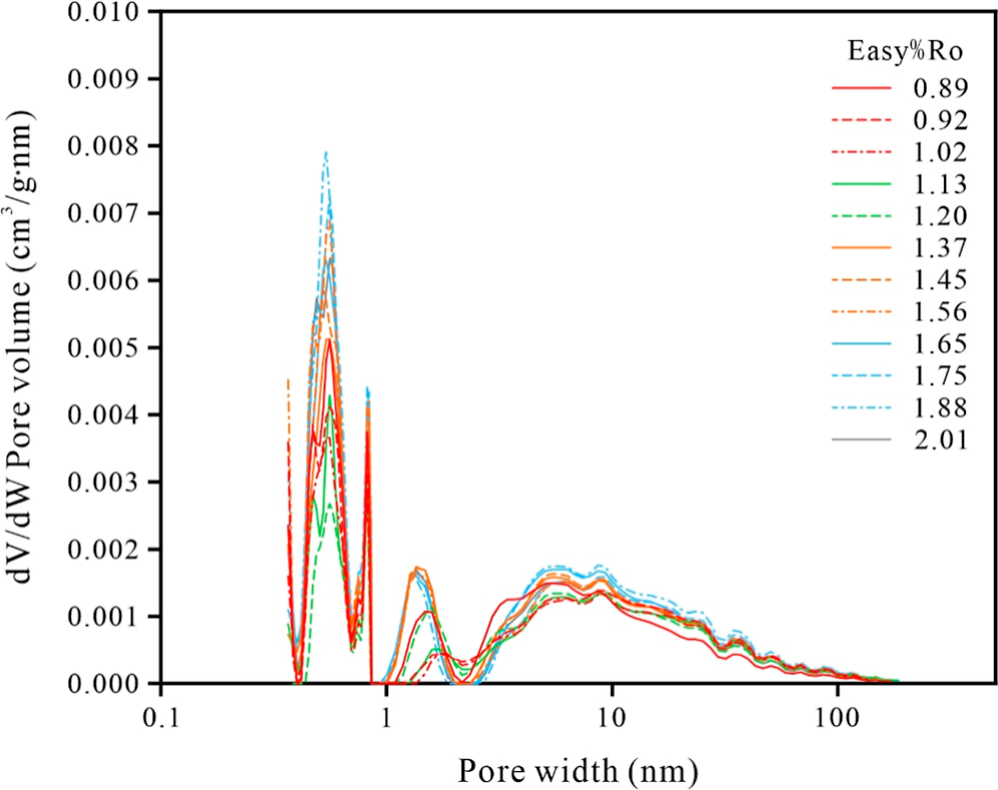
Plots of d*V*/dlog­(*W*) versus pore width for the samples.

**Figure 10:**
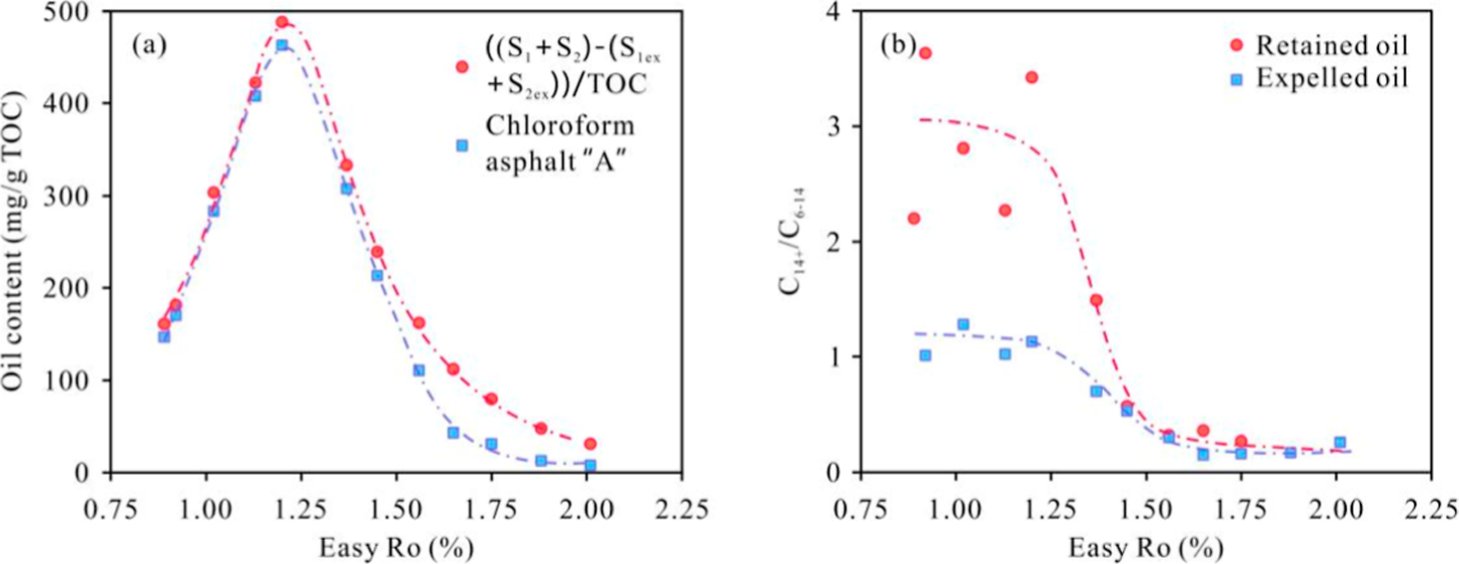
(a) Oil content of shale with maturity; (b) C_14_^+^/C_6–14_ ratio of retained oil and expelled oil with maturity.

### 5.2 Evolution of Pore Structure and Its Mechanisms

Thermal maturity plays a crucial role in the pore volume and specific surface area during thermal evolution (Figures 6–9). Hydrocarbon expulsion and production are the main factors influencing shale pore development.^8,15^ Additionally, organic acids produced during the hydrocarbon generation can influence organic pore characteristics.^54^ The pore volume does not increase linearly with increasing thermal evolution.^11,17^ In the maturity stage of 0.89%–1.20% Easy%*R*o, the volumes and specific areas of micro- and mesopores decrease, while those of macropores show an increase (Figure 11). This is likely related to the fact that the oils generated from kerogen are characterized by heavy hydrocarbons (Figures 4 and 5). These heavy hydrocarbons preferentially fill small pores, leading to a reduction in the number of micro- and mesopores. Meanwhile, the space originally occupied by kerogen is released as hydrocarbons are generated, leading to a slight increase in organic pores. These newly formed organic pores may contribute to the porosity of the shale.

**Figure 11:**
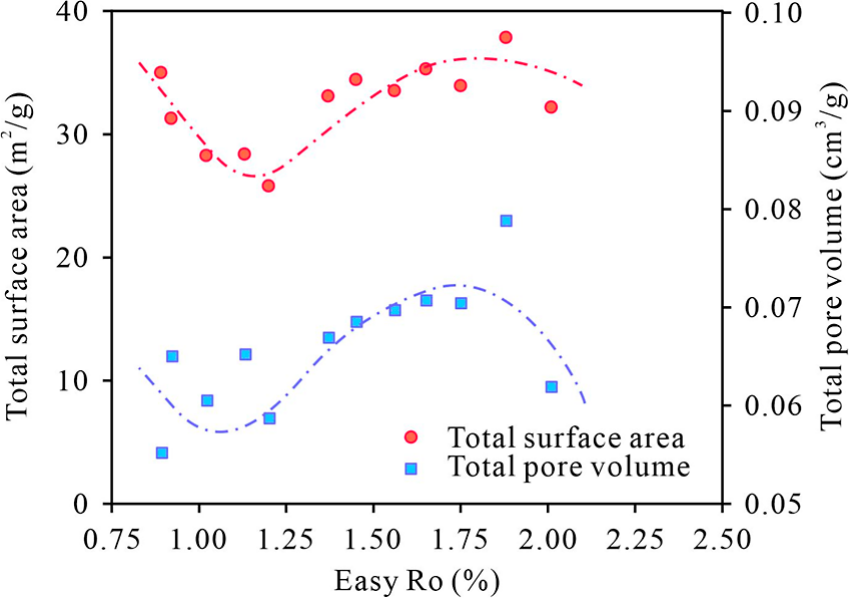
Cross-plot of Easy%*R*o against total pore volume and total surface area.

From the oil window stages to condensate oil and wet gas stages (the maturity of 1.20% to 1.88% Easy%*R*o), the volumes and specific surface areas of micro and mesopores increase, and the same trend is observed for the total pore volume and specific surface area (Figure 11). This indicates the abundant formation of secondary organic pores during this stage.^9,49,50^ The formation of new pores is attributed to the transformation of organic matter into hydrocarbons during the oil generation stage as well as the cracking of kerogen and retained oil during the gas generation stage. Furthermore, hydrocarbon gas generation gradually increases in the maturity range of 1.5%–2.0% Easy%*R*o (Figure 4a), which may lead to gas dissolution in the retained oil within shale pores. This process enhances the fluidity of the oil and facilitates the migration of hydrocarbons from smaller pores to relatively larger ones.^50–52^ The development of micropores is primarily driven by the production and expulsion of gases and light hydrocarbons during hydrocarbon generation. The changes in mesopore volume during this stage can be interpreted by several factors: first, the compaction leads to a decrease in mesopore volume;^54^ second, the depth of burial and associated high pressure and temperature conditions lead to a reduction in the pore structure;^14,29,55,56^ third, the decomposition of kerogen and retained oil, as well as hydrocarbon production during thermal evolution, leads to an increase in mesopore volume and specific surface area.^8,16^ Within the range of pore sizes of 2 nm–300 nm, the effects of load pressure and compaction during simulated experiments are minimal.^17^ In addition, the influence of liquid hydrocarbons could also be eliminated by the extraction method. In the maturity range of 1.20%–1.88% Easy%*R*o, TOC decreases (Figure 3a), and mesopore volume increases (Figure 8c). As kerogen is thermally degraded and residual oil is cracked into hydrocarbons, the space originally occupied by kerogen is released, creating a new pore space within the shale matrix. This suggests that the decomposition of organic matter and the associated carbon loss during hydrocarbon generation contribute to the formation of additional mesopores, particularly during the hydrocarbon generation stage. The total pore volume and total surface area are mainly attributed to mesopores. Meanwhile, the increase in mesopore volume is also influenced by the combined effects of hydrocarbon expulsion, which further enhances the pore connectivity and volume. During the gas generation stage (Easy%*R*o > 2.01%), specific surface areas and volumes of micro-, meso-, and macropore decrease significantly. This reduction is likely due to the massive generation and expulsion of gases, which can cause pore collapse. Additionally, the formation of coke asphalt during this stage may block pores, further reducing the pore volume and surface area.

In this study, the macropore maximum of simulated samples with different maturity stages ranges from 201.4 to 284.6 nm. Low-temperature gas adsorption experiments are unable to determine larger pore characteristics. Compared to micro-, meso-, and macropores, they exhibited the smallest changes in specific surface area, while their pore volumes fluctuate with increasing maturity. The development pattern of macropores differs from that of meso- and micropores, indicating that thermal maturity is not the sole factor influencing macropore formation. Mesopores dominate overwhelmingly the pore volume and surface area of the stimulated shale (Figure 8a), which determine the changes in total pore volume and surface area. This is consistent with the results of natural maturity samples from the Qingshankou Formation and hydrous and anhydrous simulated experiments.^57^ Mesopores contribute 76.40%–84.50% to the total volume, followed by macropores which account for 13.30%–21.20% of the total pore volume. Furthermore, mesopores make the predominant contribution to the total specific surface area, accounting for 62.80%–73.70% of the total specific surface area. Since shale oil primarily resides in mesopores, the development of mesopores plays a critical role in determining the shale oil content.

The formation of shale nanopores is influenced by various factors, with thermal maturity playing a decisive role.^11,58–60^ Additionally, the mineral composition also affects pore evolution and complexity. For example, the pores in the natural shale samples of the Triassic Yanchang Formation are predominantly associated with quartz and clay minerals,^61^ while organic pores are scarcely developed. Similar observations from the Eagle Ford Formation indicate that mineral-related pores are predominantly developed at high thermal maturity levels (*R*o = 1.20%–1.35%).^62^

The Qingshankou Formation is predominantly composed of clay-rich shale.^1^ During diagenetic evolution, quartz minerals exhibited limited alteration, while the predominant mineralogical transformation was characterized by the illitization of Illite–smectite mixed-layer (I–S) minerals. This transformation process contributes to pore development through mineral dissolution; however, subsequent precipitation of siliceous cement may partially decrease the pore. Consequently, the alteration of mineral composition exerts dual effects on pore structure modification, involving both creation and reduction of the porosity. The influence of mineral composition on porosity may be overshadowed by the effects of maturation alterations.^14^ In contrast, the thermal evolution and hydrocarbon expulsion exert significant impact on the pore volume and distribution. Thus, thermal maturity is the dominant factor governing the porosity development in shale. During thermal maturation, organic–clay complexes facilitate the development of primary organic matter-hosted pores.^41^

### 5.3 Coupled Relationship among Hydrocarbon Generation, Pore Evolution, and Geological Implications

According to the Tissot hydrocarbon generation theory,^38^ the evolution of oil content and pore structure in the Qingshankou Formation Shale can be divided into four stages. In the immature to low maturity stage (Figure 12), the cracking of kerogen into heavy hydrocarbons leads to an increase in micropore volume and a decrease in mesopore and macropore volumes.^8,54,62^ With an increasing burial depth, the shale entered the stage of thermal hydrocarbon generation (Figure 12). Organic matter underwent significant thermal decomposition, which generates residual oil and bitumen that subsequently filled micropores and mesopores, resulting in a substantial reduction in total pore volume.^11,13^ Meanwhile, hydrocarbon generation-induced pressurization and kerogen pyrolysis create dissolution pores, leading to an increase in macropores.^63,64^

**Figure 12:**
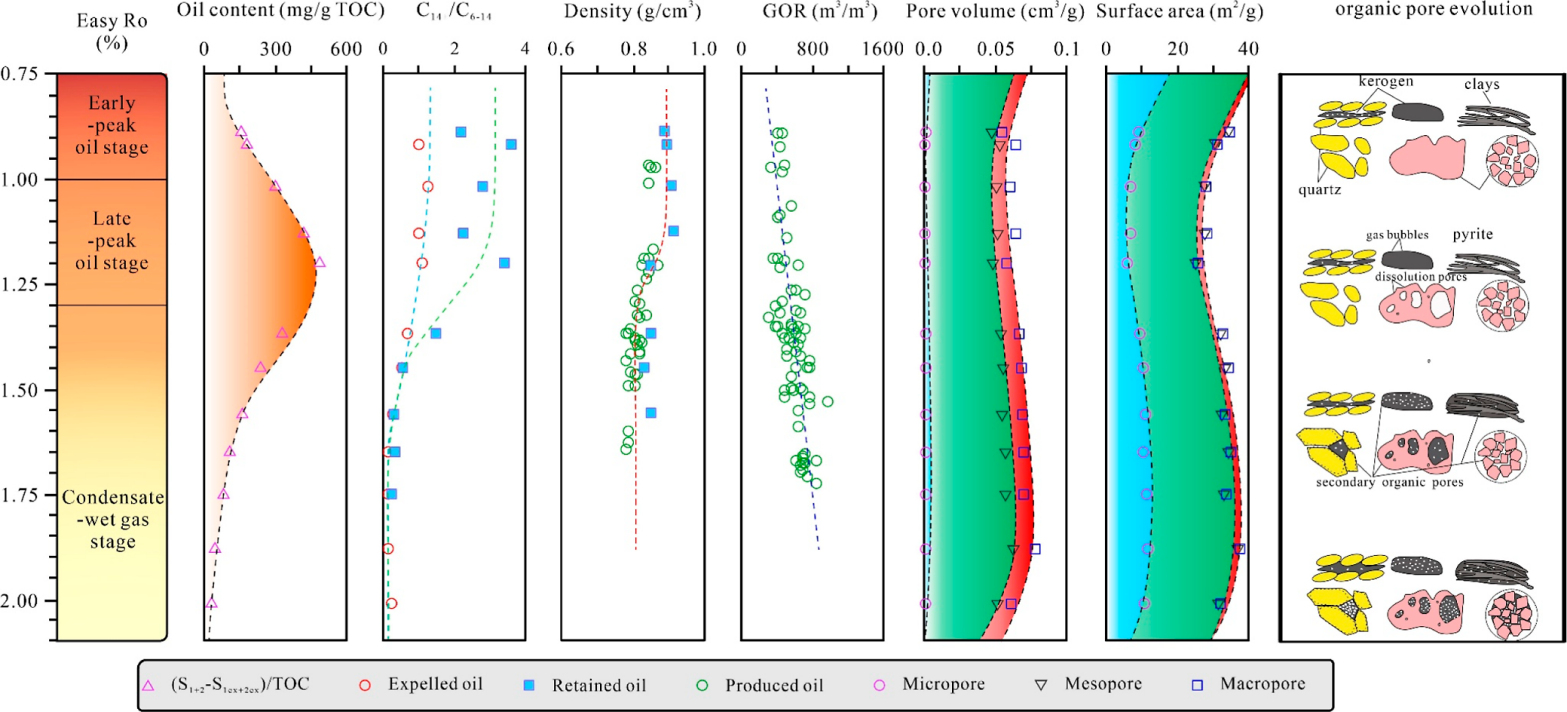
Schematic diagram illustrating the coupled evolution of oil content, hydrocarbon characteristics, and pore structure of the Cretaceous Qingshankou shale, the organic pore evolution.^50^

As organic matter maturity entered the stage of wet gas and condensate oil generation, residual oil and bitumen underwent secondary cracking to generate wet gas and condensate oil (Figure 12). This stage is characterized by high oil contents, increased gas–oil ratios, improved hydrocarbon mobility, and dominance of mesopores within organic matter,^65^ with minor development of micropores and macropores.^8,9^ Upon reaching the high to overmature stage, dry gas was generated, leading to a rapid increase in micropore and mesopore volumes, with a slight increase in macropore volumes.^13,65^ In the overmaturity stage (*R*o > 2.0%), the cessation of kerogen pyrolysis decelerates the growth of micropores and mesopores within the organic matter. Concurrently, residual bitumen underwent carbonization, causing a gradual reduction in pore volumes due to strong compaction effects.^65,66^

The Qingshankou Formation shales are mainly distributed in the northern Qijia-Gulong Sag, Sanzhao Sag, and southern Changling Sag in the Central Depression of the Songliao Basin.^24,67^ These areas are influenced by a decreased geothermal gradient.^57,59,69^ Moreover, the Qingshankou Formation shale oils have been classified into three types, i.e., interlayer, thin sandstone interlayer, and pure shale types.^1^ Integrating the results of simulation experiments in this study, we can identify the sweet spots of the Qingshankou Formation shale in different areas and types across the Songliao Basin can be identified.

The Sanzhao Sag shale is buried at shallow depths, with weak compaction effects and moderate thermal maturity ranging from 0.50% to 1.00%.^19^ This area has a low movable oil content and a high retained hydrocarbon content, making it unsuitable for the in situ development of pure shale oils. However, for interlayer shale oils, hydrocarbons are capable of migrating over short distances to the adjacent sandstone or carbonate layers.^51–53^ Similar to tight oil reservoirs, these interbedded or intercalated reservoirs can serve as the sweet spots for shale oil development in the Sanzhao Sag (Figure 13a). Additionally, they are suitable for in situ conversion of terrestrial low-maturity shale oil.^1,66^

**Figure 13:**
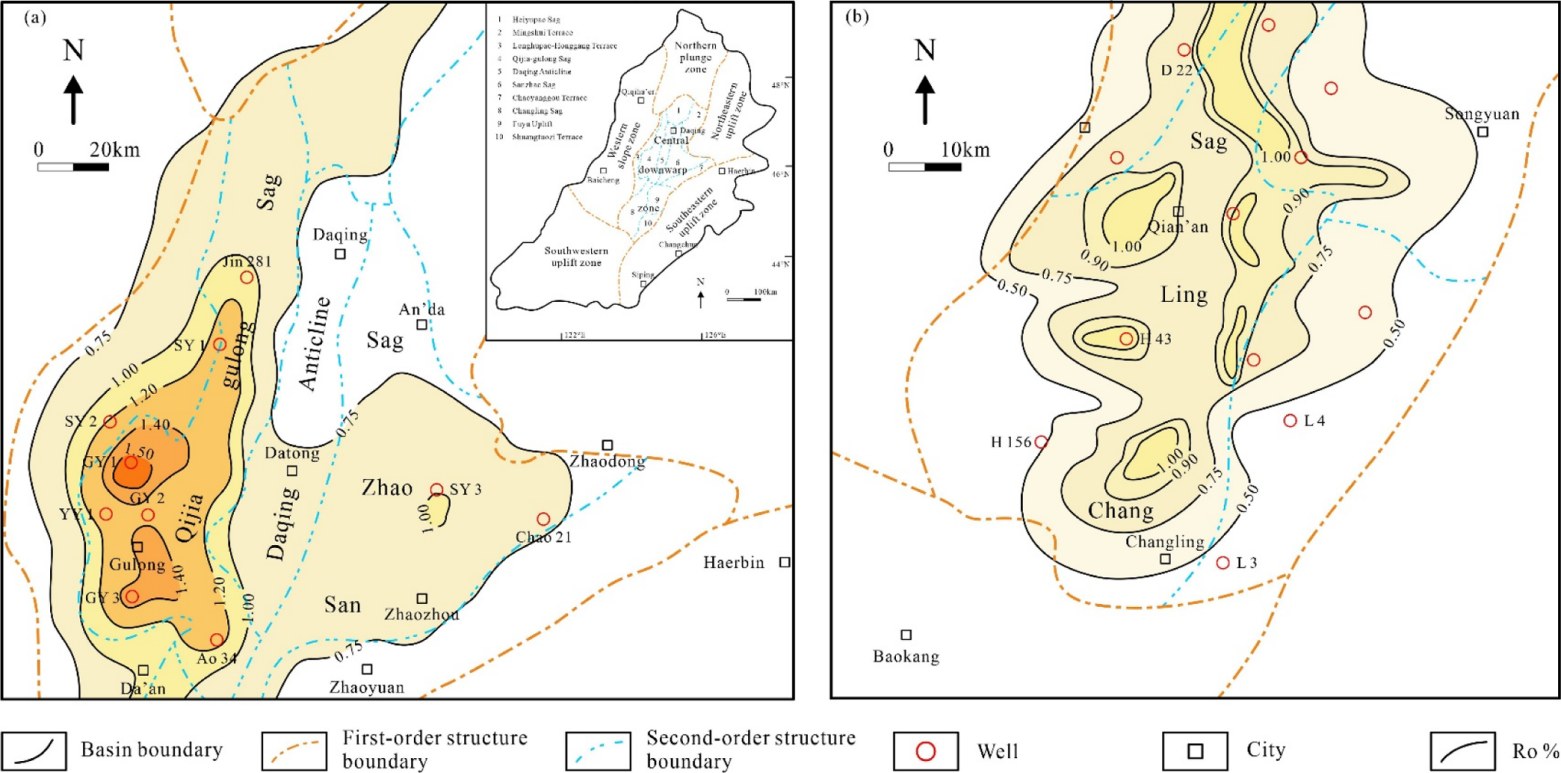
*R*o isogram of the Qingshankou Formation shale in the central depression of the Songliao Basin. (a) The Qijia-Gulong Sag and Sanzhao Sag.^21^ (b) The Changling Sag.^69^

The Qijia-Gulong Depression has suitable burial depth and thermal maturity (1.20%–1.67%).^1^ Hydrocarbons generated here are predominantly light components with high movable oil contents. Moreover, hydrocarbon generation led to an increase in the porosity of the shale. As a result, shale within this maturity range becomes the most conducive target for exploration and development (Figure 13). In the Changling Sag, the maturity ranges from 0.8% to 1.20%.^67^ This can be divided into two sweet spot sections: (1) 0.8% < *R*o < 1.0%, which is favorable for interbedded or interlayer shale oil development, and (2) 1.0% < *R*o < 1.20%, which is suitable for pure shale oil exploration and development (Figure 13b).

## 6 Conclusions

In this study, we conducted hydrocarbon generation simulation experiments on low-maturity Qingshankou shale. We carried out detailed analyses of the oil content and pore characteristics of the simulated shales at different thermal maturity stages. Furthermore, we explored the coupled relationship between organic matter generation and pores during the thermal evolution process.

The thermal evolution significantly affects both the composition and fluidity of retained hydrocarbons and the distribution of micro- and mesopores. Mesopores constitute the dominant pore type in the Qingshankou Formation Shale during different maturity stages. When kerogen thermal degradation is the dominant process (Easy%*R*o < 1.20%), the generated hydrocarbons are predominantly heavy compounds, characterized by a low gas–oil ratio and poor fluidity, and hydrocarbon plugging reduces pore volume and specific surface area. The peak of hydrocarbon generation occurs at Easy%*R*o of 1.20%, with a maximum oil generation of 500 mg/g TOC. Within the thermal maturity range of 1.20%–1.60 *R*o %, the retained hydrocarbons in shale are mainly light oil, with increased porosity, high gas–oil ratio, and good intralayer shale oil mobility. When thermal cracking of retained hydrocarbons becomes the dominant process (Easy%*R*o > 1.60%), the retained hydrocarbons generate a large amount of natural gas, and the hydrocarbon content is very low of the shale layer. Meanwhile, the retained hydrocarbons undergo a significant reduction. Both pore volume and specific surface area seem to reduce, which primarily results from hydrocarbon plugging effects and pore collapse induced by hydrocarbon pressurization.

Based on the coupled evolution of oil content and pore structure in this study, these characteristics facilitate the exploitation of pure shale-type oil within the thermal maturity range of 1.20%–1.60 *R*o %, particularly in the Qijia-Gulong Depression and the Changling Sag. In contrast, the medium-to-low maturity shales in the Sanzhao Sag and the Changling Sag are favorable for interbedded or intercalated shale oil, making them suitable targets for in situ conversion of medium–low-maturity lacustrine shale oil.^68^
